# A cohort study of reproductive and hormonal factors and renal cell cancer risk in women

**DOI:** 10.1038/sj.bjc.6603629

**Published:** 2007-02-20

**Authors:** G C Kabat, S A Navarro Silvera, A B Miller, T E Rohan

**Affiliations:** 1 Department of Epidemiology and Population Health, Albert Einstein College of Medicine, 1300 Morris Park Avenue, Room 1301, NY 10461, USA; 2 Department of Public Health Sciences, University of Toronto, Toronto, Canada

**Keywords:** kidney neoplasms, cohort, reproductive factors, hormones, smoking, body mass index, parity

## Abstract

We examined the association of reproductive and hormonal factors with renal cell cancer risk in a cohort study of 89 835 Canadian women. Compared with nulliparous women, parous women were at increased risk (hazard ratio (HR) 1.78, 95% confidence interval (CI) 1.02–3.09), and there was a significant gradient of risk with increasing levels of parity: relative to nulliparous women, women who had ⩾5 pregnancies lasting 4 months or more had a 2.4-fold risk (HR=2.41, 95% CI=1.27–4.59, *P* for trend 0.01). Ever use of oral contraceptives was associated with a modest reduction in risk. No associations were observed for age at first live birth or use of hormone replacement therapy. The present study provides evidence that high parity may be associated with increased risk of renal cell cancer, and that oral contraceptive use may be associated with reduced risk.

Incidence rates of renal cell cancer have been increasing both in the USA and globally since the 1970s (Chow *et al*, 1999; McLaughlin *et al*, 2006), and renal cell carcinomas account for approximately 80% of renal cancers (McLaughlin *et al*, 2006). While cigarette smoking, obesity, and hypertension are established risk factors for renal cell cancer, few other risk factors have been identified conclusively (Lindblad and Adami, 2002; McLaughlin *et al*, 2006). Smoking, obesity, and hypertension have been estimated to account for roughly 68% of renal cancer incidence in women (Benichou *et al*, 1998), leaving a substantial proportion of this cancer still unexplained.

A number of findings suggest a possible role of reproductive or hormonal factors in renal cell cancer. First, over the past 30 years, incidence rates of renal cell cancer have been increasing more rapidly in women than in men in the USA and the UK (Katz *et al*, 1994; Chow *et al*, 1999; Tate *et al*, 2003), suggesting a possible association with exposure to exogenous hormones. Second, steroid hormone receptors have been found in both normal and cancerous renal cell tissue, suggesting hormonal regulation (Ronchi *et al*, 1984; Siiteri, 1987; Vugrin, 1987). Third, renal tumours can be induced in experimental animals with diethylstilbestrol and oestradiol (Kirkman, 1959; Reznik-Schuller, 1979; Li and Li, 1990). Finally, obesity, a consistent risk factor for renal cell cancer, is the main source of endogenous exposure to oestrogen in postmenopausal women (Siiteri, 1987).

Only a few studies have examined endocrine factors in relation to renal cell cancer risk (Krieger *et al*, 1992; McCredie and Stewart, 1992; Cantor *et al*, 1993; Mellemgaard *et al*, 1994; Chow *et al*, 1995; Lindblad *et al*, 1995; Gago-Dominguez *et al*, 1999; McLaughlin and Lipworth, 2000; Lambe *et al*, 2002; Nicodemus *et al*, 2004) and their findings have been inconsistent. Only two cohort studies (Lambe *et al*, 2002; Nicodemus *et al*, 2004) have addressed the role of reproductive and hormonal factors, but each was limited to two exposures of interest. Given the current lack of data from prospective studies on this topic, we examined the association between reproductive and hormonal factors and renal cell cancer risk in a cohort of Canadian women.

## MATERIALS AND METHODS

### Study population

The study, which has been described in detail elsewhere (Miller *et al*, 1992), was conducted among participants in the Canadian National Breast Screening Study (NBSS), a randomised controlled trial of screening for breast cancer. A total of 89 835 women aged 40–59 years with no history of breast cancer were recruited into the trial between 1980 and 1985. Participating women were randomised to receive either (1) screening with annual mammography, breast physical examination, and instruction on breast self-examination on four or five occasions, or (2) community care after a single breast physical examination and instruction on breast self-examination. The NBSS was approved by the appropriate Institutional Review Boards, and informed consent was obtained from all study participants.

### Questionnaires and anthropometric measurements

At recruitment into the cohort, participants completed self-administered questionnaires that sought information on demographic characteristics, lifestyle factors (including cigarette smoking), menstrual and reproductive history, and use of oral contraceptives and hormone replacement therapy (HRT). Specifically, participants were asked questions about their age at menarche, menopausal status, number of pregnancies lasting greater than 4 months (parity), age at first live birth, and ever use and duration of use of oral contraceptives and of HRT. In addition, height and weight were measured at the initial physical examination.

### Ascertainment of incident renal cell cancer cases and deaths

Incident cases of renal cell cancer and deaths from all causes were ascertained, respectively, by means of computerised record linkages to the Canadian Cancer Database and to the National Mortality Database, both of which are maintained by Statistics Canada. The linkages to the databases yielded data on cancer incidence and mortality to 31 December 2000 for women in Ontario, 31 December 1998 for women in Quebec, and 31 December 1999 for women in other provinces.

### Statistical analysis

Of the 89 835 women recruited into the study, we excluded women with prevalent renal cancer at baseline (*n*=14), leaving 89 821 women available for analysis, among whom 199 incident cases of renal cancer were observed. We further excluded 27 cases with cancer of the renal pelvis and a morphology code of transitional cell carcinoma, leaving 172 cases with cancer of the renal parenchyma. For these analyses, study participants were considered at risk from their date of enrolment until the date of diagnosis of their renal cell carcinoma, termination of follow-up (the date to which cancer incidence data were available for women in the corresponding province) or death, whichever occurred first. Cox proportional hazards models (using age as the time scale) were used to estimate hazard ratios (HRs) and 95% confidence intervals (CIs) for the association between reproductive and hormonal factors and renal cell cancer risk. All multivariate models included terms for body mass index (BMI) (kg m^−2^) (quintiles), pack-years of smoking (never smokers+five levels), menopausal status at baseline (pre-, peri-, postmenopausal), education (three levels), study centre, and randomisation group (intervention or control in the original clinical trial). Women who reported having regular menstrual periods within the 12 months before baseline or who had had a hysterectomy without bilateral oophorectomy and were less than 45 years of age were classified as premenopausal. Women whose menstrual periods ceased more than 12 months before baseline, those who had had a bilateral oophorectomy, and those who had had a hysterectomy and were over 55 years of age were considered postmenopausal (Rohan *et al*, 1998). Women whose periods were irregular and those who had a hysterectomy and were between 45 and 55 years old were considered perimenopausal. To test for trends in risk with increasing levels of the exposures of interest, we either assigned the categorical variables (parity) their ordinal number or the category median (age at first live birth, duration of oral contraceptive use, duration of HRT) and then fitted the assigned value of each risk factor as a continuous variable in the risk models. We then evaluated the statistical significance of the corresponding coefficient using the Wald test (Rothman and Greenland, 1998). Tests for interaction were based on comparing the likelihood ratio tests in models with and without the product terms representing the variables of interest. The likelihood ratio test that all of the interaction parameters were 0 was performed by referring 2^*^ the absolute difference in the log likelihoods of models with and without interaction terms to the *χ*^2^ distribution with degrees of freedom equal to the number of interaction parameters. Use of the life-test procedure in SAS™ showed that the proportional hazards assumption was met in this data set. All analyses were performed using SAS version 9 (SAS Institute Cary, NC, USA).

## RESULTS

The average duration of follow-up for cohort members was 16. 4 years (1 474 006 person-years). Table 1 shows the baseline characteristics of the study population by case status at the end of follow-up. Briefly, compared to non-cases, renal cell cancer cases were older, had a higher mean BMI, were more likely to have had five or more pregnancies lasting greater than 4 months, were more likely to be postmenopausal, were more likely to be current smokers, and were less likely to have ever used oral contraceptives.

Compared to nulliparous women, parous women had an increased risk of renal cell cancer (multivariate-adjusted HR 1.78, 95% CI 1.02–3.09), and there was a significant gradient with increasing level of parity: relative to nulliparous women, women who had ⩾5 pregnancies lasting 4+ months had a 2.4-fold risk (HR=2.41, 95% CI=1.27–4.59, *P* for trend=0.01) (Table 2). A relatively late age at menarche appeared to be associated with a modestly increased risk, and ever use of oral contraceptives was associated with slightly reduced risk. However, these results did not achieve statistical significance, and there was no clear trend with age at menarche or duration of oral contraceptive use. Perimenopausal and postmenopausal women were at increased risk compared to premenopausal women. No associations were seen with age at first live birth or use of HRT.

Both smoking and BMI showed positive dose–response relationships, in line with previous studies. Compared to never smokers, smokers of 40+ pack-years had a HR of 2.12, 95% CI 1.21–3.71, *P* for trend 0.004; compared to the lowest quintile of BMI, women in the highest quintile had a HR of 1.76, 95% CI 1.03–3.03, *P* for trend 0.02. Table 3 presents the associations of parity and oral contraceptive use stratified by major known risk factors. The positive association of parity with renal cell cancer was limited to women 50–59 years old at enrolment (*P* for interaction=0.11) and to never smokers (*P* for interaction <0.005). For women aged 50–59 years at baseline, the HR associated with five or more pregnancies lasting 4+ months was 3.43, 95% CI 1.40–8.43, *P* for trend 0.009, and a HR of 3.72, 95% CI 1.35–10.24, *P* for trend 0.0006 was observed among never smokers. The association of parity with renal cell cancer did not differ by the level of BMI. The inverse association of oral contraceptive use with renal cell cancer was more pronounced among never smokers: HR 0.55, 95% CI 0.34–0.88 (*P* for interaction 0.01). There was also a suggestion that the associations for oral contraceptive use were stronger in younger women (40–49 years old) and overweight women (BMI⩾25.1 kg m^−2^).

## DISCUSSION

In the prospective study reported here, we found a statistically significant increased risk of renal cell cancer in women with increasing parity, which was comparable in strength to the associations with smoking and BMI. The association with parity was strongest in women who were older at enrolment and in never smokers. There was a suggestion of reduced risk with a relatively early age at menarche and with ever use of oral contraceptives, but these associations did not achieve statistical significance. Renal cell cancer risk was not associated with age at first live birth or use of HRT. Both smoking and BMI showed positive dose–response relationships with renal cancer risk in this cohort, in line with previous studies.

Most previous studies have examined only a limited number of reproductive factors, most commonly parity and age at first live birth. Of nine studies that provided data on parity, four found no association (Krieger *et al*, 1992; McCredie and Stewart, 1992; Mellemgaard *et al*, 1994; Gago-Dominguez *et al*, 1999), one showed a borderline positive association (Cantor *et al*, 1993), and four (including the two cohort studies) showed a significant positive association (Chow *et al*, 1995; Lindblad *et al*, 1995; Lambe *et al*, 2002; Nicodemus *et al*, 2004). Similar to these latter studies, we found a significant positive dose–response relationship with increasing parity.

Findings regarding an association of age at first live birth have been weaker and less consistent. Two case–control studies indicated no association of age at first live birth with renal cell cancer (Cantor *et al*, 1993; Lindblad *et al*, 1995), whereas two other case–control studies suggested a borderline inverse association (Mellemgaard *et al*, 1994; Chow *et al*, 1995). The large cohort study by Lambe *et al* (2002) reported a significant inverse association, the OR in the highest quartile relative to that in the lowest being 0.89 (95% CI 0.69–1.15), *P* for trend 0.04, suggesting, at most, an association of relatively small magnitude. We found no clear evidence of a trend with age at first live birth, and the 40% reduction in risk among women who had their first live birth at 30 years or greater was based on only 10 cases.

Of the four studies (all case–control) presenting findings on age at menarche, two (McCredie and Stewart, 1992; Chow *et al*, 1995) showed no association, whereas the other two suggested that risk decreases with increasing age at menarche (Mellemgaard *et al*, 1994; Lindblad *et al*, 1995); however, neither the individual risk estimates nor the trends approached statistical significance. We found a nonsignificant increased risk with older age at menarche, although the trend was not statistically significant. Analysis of the large case–control study by Lindblad *et al* (1995) showed that, after careful adjustment for BMI, parity, and other factors, the inverse associations with age at first birth and age at menarche were attenuated.

Of four studies of oral contraceptive use (Mellemgaard *et al*, 1994; Chow *et al*, 1995; Lindblad *et al*, 1995; Gago-Dominguez *et al*, 1999), three showed some evidence of reduced risk among users, the other finding no association (Gago-Dominguez *et al*, 1999). Our results are suggestive of a 20% reduction in risk among ever users of oral contraceptives, although there was no trend with duration of use. In the analysis stratified by age at enrolment, a greater reduction in risk was seen in younger women. Findings concerning hormone replacement use have been inconsistent (Mellemgaard *et al*, 1994; Chow *et al*, 1995; Lindblad *et al*, 1995; Gago-Dominguez *et al*, 1999; Nicodemus *et al*, 2004). In keeping with two case–control studies (Lindblad *et al*, 1995; Gago-Dominguez *et al*, 1999), we found no evidence of an association with ever use or with duration of use.

Whereas smoking, obesity, and hypertension are established risk factors for renal cell cancer (Lindblad and Adami, 2002; McLaughlin *et al*, 2006), it is unclear whether reproductive or hormonal factors play a role. A number of findings support the concept that pregnancy-associated hormonal changes, and, particularly, high oestrogen levels, may promote malignant changes by stimulating renal cell proliferation either directly or via growth factors (Concolino *et al*, 1993). These findings include the fact that administration of potent oestrogens has been shown to induce renal cancers in the Syrian hamster (Kirkman, 1959; Reznik-Schuller, 1979; Li and Li, 1990; Cavalieri *et al*, 2001), the presence of oestrogen and progesterone receptors in normal and malignant renal cells (Concolino *et al*, 1976; Ronchi *et al*, 1984), and the observed association with obesity, which provides a major source of oestrogen in postmenopausal women. However, the evidence supporting a role of exposure to endogenous or exogenous oestrogens in renal cell carcinogenesis is conflicting. The association of factors such as age at menarche, age at menopause, parity, and exogenous oestrogen exposure with renal cell cancer presents a markedly different pattern from that seen for breast and endometrial cancer. The male predominance in incidence rates of the disease, the positive association with parity in some studies, and the observed reduction in risk in users of oral contraceptives in some studies suggest that exposure to endogenous oestrogens may be protective rather than a risk factor. Furthermore, the appropriateness of the Syrian hamster animal model to renal cell carcinoma in humans has been questioned (Gago-Dominguez *et al*, 2002).

The positive association with parity may reflect the association of normal pregnancies with hyperfiltration (Krutzen *et al*, 1992; Sturgiss *et al*, 1996). Based on animal studies, glomerular hyperfiltration may play a role in the development of glomerulosclerosis (Neuringer and Brenner, 1992; Westenend *et al*, 1992), which makes the nephrons more susceptible to exposure to carcinogens. Furthermore, pregnancy-associated weight gain could provide a partial explanation for the association of parity with renal cell cancer (Lambe *et al*, 2002).

Recently, an attempt has been made to provide a unified explanation of the aetiology of renal cell cancer based on the mechanism of lipid peroxidation (Gago-Dominguez *et al*, 2002). Gago-Dominguez *et al* noted that lipid peroxidation is increased among both obese and hypertensive subjects, and that in animal models lipid peroxidation of the proximal renal tubules has been shown to be a necessary condition for chemically induced renal carcinogenesis. The authors argued that lipid peroxidation may also explain the association of other risk factors, including cigarette smoking, the male preponderance of kidney cancer, parity, diabetes, and a protective effect of antioxidants, as there is evidence indicating that these risk factors are associated with increased lipid peroxidation, and that dietary antioxidants are associated with decreased lipid peroxidation. In keeping with this, pregnant women have been observed to have higher levels of serum lipid peroxide compared to nonpregnant women (Gago-Dominguez *et al*, 2002). Gago-Dominguez *et al* (2002) further point to Fe-NTA-induced renal carcinogenesis in rats and mice as providing an experimental model relevant to human renal cell cancer. Intraperitoneal injections of Fe-NTA in rodents induce necrosis of the proximal renal tubules and, subsequently, a high incidence (60–92%) of renal cell carcinoma. This model appears to correspond to human renal cell carcinoma, given that the tumour incidence in male rats is twice as high as in female rats, and that the histology involves clear and granular cells.

Strengths of the present study include its prospective nature, the availability of information on a number of reproductive and hormonal factors as well as other risk factors, and the high level of follow-up over a period of 16 years. A number of limitations should also be noted. First, information was not available on several factors of interest, including history of hypertension, miscarriages and abortions, and the specific regimen of HRT. Second, only information collected at baseline was available, so that we were not able to take into account changes in the exposures of interest over the follow-up period. Nevertheless, we were able to detect monotonic dose–response relationships with smoking and BMI, and our results regarding parity and oral contraceptive use are in agreement with the findings of the larger previous studies. It is unclear, however, whether changes in parity or in oral contraceptive use have contributed to the increasing rates of renal cell carcinoma in women.

## Figures and Tables

**Table 1:** Baseline characteristics of the study population by outcome

**Factor**	**Incident renal cell cancer cases (*n*=172)**	**Non-cases (*n*=89 617)**
Mean age at baseline (years)	50.7 (5.6)	48.5 (5.6)
Mean BMI (kg m^−2^)^a^	26.1 (5.1)	25.1 (4.7)
Mean age at menarche (years)	13.0 (1.4)	12.8 (2.2)
Mean age at first live birth (years)^b^	23.7 (3.7)	24.2 (4.8)

*Parity* (%)		
Nulliparous	9.3	14.4
1–2	32.0	35.5
3–4	40.1	38.4
5+	18.6	11.7
Postmenopausal (%)	44.2	37.0

*Cigarette smoking* (%)		
Never smoked	44.8	52.2
Former smoker	25.0	26.0
Current smoker	30.2	21.8
HRT use (% ever)^c^	46.1	47.0
Oral contraceptives use (% ever)	51.7	58.1

a BMI=body mass index.

b Among parous women only.

c HRT=hormone replacement therapy; results among postmenopausal women only.

**Table 2:** Age-adjusted and multivariate-adjusted hazard ratios (HR) and 95% confidence intervals (95% CI) for the association between lifestyle, reproductive, and hormonal factors and risk of incident renal cell cancer

**Factor**	**Cases/person years**	**Age-adjusted HR^a^ (95% CI)**	**Multivariate HR^b^ (95% CI)**
*Parity*			
Nulliparous	16/212 504	1.00 (reference)	1.00 (reference)
Parous	156/1 261 502	1.63 (0.97–2.72)	1.78 (1.02–3.09)
1–2	55/522 816	1.40 (0.80–2.44)	1.62 (0.90–2.92)
3–4	69/567 220	1.61 (0.93–2.78)	1.77 (0.99–3.16)
5+	32/171 465	2.48 (1.36–4.53)	2.41 (1.27–4.59)
*P* for trend		0.002	0.01

*Age at first live birth (years)* c			
<20	23/212 504	1.00 (reference)	1.00 (reference)
20–22	40/469 625	1.77 (1.02–3.07)	0.88 (0.52–1.48)
23–25	46/360 111	1.68 (0.95–2.97)	1.00 (0.59–1.69)
26–29	37/273 117	1.79 (1.00–3.22)	1.09 (0.63–1.90)
30+	10/151 912	0.87 (0.40–1.93)	0.54 (0.25–1.17)
*P* for trend		0.97	0.45

*Age at menarche (years)*			
<12	25/257 779	1.00 (reference)	1.00 (reference)
12	39/341 764	1.19 (0.72–1.96)	1.21 (0.73–2.00)
13	52/429 850	1.25 (0.78–2.02)	1.33 (0.82–2.15)
14+	56/445 932	1.32 (0.83–2.12)	1.37 (0.85–2.20)
*P* for trend		0.25	0.20

*Menopausal status*			
Pre-	61/725 258	1.00 (reference)	1.00 (reference)
Peri-	35/209 540	1.98 (1.30–3.01)	1.74 (1.12–2.71)
Post-	76/539 649	1.67 (1.19–2.34)	1.47 (1.00–2.16)

*Oral contraceptives*			
Never	83/611 469	1.00 (reference)	1.00 (reference)
Ever	89/863 819	0.75 (0.56–1.02)	0.80 (0.58–1.09)
1–11 months	19/170 748	0.82 (0.50–1.35)	0.81 (0.48–1.35)
12–35 months	21/194 388	0.80 (0.49–1.29)	0.86 (0.53–1.40)
36–95 months	29/298 430	0.71 (0.47–1.09)	0.75 (0.49–1.17)
96+ months	29/200 252	0.73 (0.45–1.20)	0.80 (0.48–1.31)
*P* for trend		0.08	0.20

*HRT use* d			
Never	122/1 102 965	1.00 (reference)	1.00 (reference)
Ever	50/372 361	1.24 (0.89–1.72)	0.98 (0.69–1.41)
1–12 months	18/118 416	1.39 (0.85–2.28)	1.02 (0.56–1.85)
13–59 months	12/106 984	1.04 (0.57–1.87)	0.98 (0.53–1.82)
60+ months	13/104 848	1.32 (0.80–2.17)	0.91 (0.51–1.63)
*P* for trend		0.25	0.74

a Adjusted for age (time to event variable).

b Adjusted for age (time to event variable), pack-years (never smokers + five levels), body mass index (quintiles), menopausal status (pre-, peri-, postmenopausal), education (three levels), study centre, randomisation group (intervention *vs* control), and the other variables in the table.

c Parous women only.

d Postmenopausal women only.

**Table 3:** Multivariate-adjusted hazard ratios (HR) and 95% confidence intervals (95% CI) for the association between parity and oral contraceptive use and renal cell cancer, stratified by other risk factors

**Stratification variables**	**HR^a^ (95% CI)**	**HR^a^ (95% CI)**	***P* for interaction**
**Age at enrolment**	**40–49 years**	**50–59 years**	
*Parity*			
Nulliparous	1.00 (reference)	1.00 (reference)	
1–2	1.05 (0.90–1.28)	2.21 (0.92–5.33)	
3–4	1.15 (0.53–2.48)	2.28 (0.96–5.41)	
5+	0.97 (0.32–2.96)	3.43 (1.40–8.43)	0.11
*P* trend	0.86	0.009	

*Oral contraceptive use*			
Never	1.00 (reference)	1.00 (reference)	
Ever	0.77 (0.46–1.29)	0.90 (0.61–1.34)	0.35

**Smoking status**	**Never smokers**	**Ever smokers**	
*Parity*			
Nulliparous	1.00 (reference)	1.00 (reference)	
1–2	1.40 (0.51–3.84)	1.46 (0.73–2.95)	
3–4	2.71 (1.06–6.97)	1.10 (0.53–2.27)	
5+	3.72 (1.35–10.24)	1.65 (0.72–3.78)	<0.005
*P* for trend	0.0006	0.67	

*Oral contraceptive use*			
Never	1.00 (reference)	1.00 (reference)	
Ever	0.55 (0.34–0.88)	0.97 (0.64–1.49)	0.01

**Body mass index**	**<25.1 kg m^−2^**	**>25.1 kg m^−2^**	
*Parity*			
Nulliparous	1.00 (reference)	1.00 (reference)	
1–2	1.34 (0.63–2.86)	1.45 (0.71–3.65)	
3–4	1.47 (0.70–3.11)	1.61 (0.71–3.65)	
5+	2.07 (0.86–5.01)	2.13 (0.88–5.11)	0.48
*P* for trend	0.12	0.09	

*Oral contraceptive use*			
Never	1.00 (reference)	1.00 (reference)	
Ever	0.87 (0.55–1.37)	0.77 (0.50–1.19)	0.63

a Adjusted for age (time to event variable), menopausal status (pre-, peri-, postmenopausal), education (three levels), study centre, randomisation group (intervention *vs* control), and the other variables in the table apart from those involved in a particular interaction.
